# Drinking Motives As Mediators of the Associations between Reinforcement Sensitivity and Alcohol Misuse and Problems

**DOI:** 10.3389/fpsyg.2016.00718

**Published:** 2016-05-24

**Authors:** Joseph Studer, Stéphanie Baggio, Marc Dupuis, Meichun Mohler-Kuo, Jean-Bernard Daeppen, Gerhard Gmel

**Affiliations:** ^1^Alcohol Treatment Centre, Lausanne University Hospital CHUVLausanne, Switzerland; ^2^Life Course and Social Inequality Research Centre, University of LausanneLausanne, Switzerland; ^3^Institute of Psychology, University of LausanneLausanne, Switzerland; ^4^Epidemiology, Biostatistics and Prevention Institute, University of ZurichZurich, Switzerland; ^5^Addiction SwitzerlandLausanne, Switzerland; ^6^Center for Addiction and Mental HealthToronto, ON, Canada; ^7^Alcohol and Health Research Unit, University of the West of EnglandBristol, UK

**Keywords:** BIS/BAS scales, risky single occasion drinking, alcohol use disorder, DMQR-SF, young adults

## Abstract

Alcohol may be used and misused for different reasons, i.e., to enhance positive affect and to cope with negative affect. These to pathways are thought to depend on two distinct and relatively stable neurobiological systems: the behavioral activation (BAS; i.e., fun seeking, drive, reward responsiveness) and behavioral inhibition (BIS) systems. This study investigates the associations of BAS and BIS sensitivity with risky single-occasion drinking and alcohol use disorder in a representative sample of 5362 young Swiss men. In order to better understand the contribution of more proximal motivational factors in the associations of BIS and BAS with alcohol outcomes, mediations via drinking motives (i.e., enhancement, social, coping, conformity) was also tested. Risky single-occasion drinking and alcohol use disorder were positively associated with fun seeking and negatively with reward responsiveness. Drive was negatively associated with risky single-occasion drinking. BIS was positively associated with alcohol use disorder and negatively with risky single-occasion drinking. Positive associations of fun seeking with risky single-occasion drinking and alcohol use disorder were partially mediated mainly by enhancement motives. Negative association of drive with risky single-occasion drinking was partially mediated by conformity motives. The negative reward responsiveness—alcohol use disorder association was partially mediated, whereas the negative reward responsiveness—risky single-occasion drinking association was fully mediated, mainly by coping and enhancement motives. The positive BIS–alcohol use disorder association was fully mediated mainly by coping motives. Fun seeking constitutes a risk factor, whereas drive and reward responsiveness constitute protective factors against alcohol misuse and disorder. BIS constitutes a protective factor against risky single-occasion drinking and a risk factor for alcohol use disorder. The results of the mediation analysis suggest that prevention strategies targeting coping and enhancement motives may reduce the risk associated with high BIS and with high fun seeking, respectively.

## Introduction

### Alcohol misuse and alcohol use disorder in young adults

Alcohol use, particularly heavy drinking, is one of the most important risk factor for mortality and morbidity (Rehm et al., 2006) and thus constitutes a major concern for public health researchers. Young adults are particularly vulnerable since risky patterns of alcohol use such as risky single-occasion drinking (RSOD; also called binge drinking or heavy episodic drinking) and prevalence of alcohol use disorder (AUD) peak in early adulthood (Gmel et al., 2013; Grant et al., 2015). RSOD is a pattern of alcohol use defined as heavy use over a short period of time, e.g., drinking about 60 g of pure ethanol or more on a single occasion (Gmel et al., 2011). The most common negative consequences of RSOD include the acute physiological and behavioral effects of excessive alcohol use such as blackouts, regretted actions, violence, accidents, or injuries (Wechsler et al., 1994; Daeppen et al., 2005; Kuntsche and Gmel, 2013). Although, RSOD and AUD are related (Knight et al., 2002; Baggio et al., 2015), they are not the same. The diagnostic and statistical manual of mental disorders fifth edition (DSM-5; American Psychiatric Association, 2013) defines AUD as a problematic pattern of using alcohol that results in impairment in daily life or noticeable distress. Thus, RSOD, often occurring on weekends (Gmel et al., 2008), must not necessarily result in impairment in daily life, whereas AUD, being often related to a more regular heavy drinking over time (Rehm et al., 2013), does. RSOD may lead to AUD, but not all risky single occasion drinkers necessarily develop AUD though AUD is commonly accompanied by heavy drinking occasions, satisfying the criterion of 60 g of pure ethanol or more on a single occasion (Gmel et al., 2011). Therefore, a better understanding of the risk factors associated with RSOD and AUD is needed in order to develop more efficient prevention strategies.

### Theories of motivation and reinforcement sensitivity that may impact alcohol misuse and alcohol use disorder

Motivational models of alcohol use postulated the existence of two distinct reinforcement pathways playing a major role in the development and maintenance of alcohol use and misuse (Cooper et al., 1995; Cox and Klinger, 2011). Some individuals drink alcohol for its positive reinforcing properties, in order to increase their positive affective experience (e.g., mood enhancement). Others drink alcohol for its negative reinforcing properties, in order to dampen their negative emotions and cope with distress and anxiety (tension reduction). These two reinforcement pathways are thought to result from different personality characteristics.

Reinforcement Sensitivity Theory (RST; Gray, 1972, 1982) considers that behaviors fundamentally consist of two core motivational systems underlying sensitivity to reward and sensitivity to punishment, i.e., the Behavioral Activation System (BAS) and the Behavioral Inhibition System (BIS). BAS is sensitive to signals of reward and relief of punishment, and is thought to control appetitive motivation oriented toward reward (Corr, 2008). According to Carver and White (1994), BAS has three facets: drive, i.e., the persistent pursuit of desired goals; fun seeking, i.e., the desire for new rewards and the willingness to approach a potentially rewarding event on the spur of the moment; and reward responsiveness, i.e., positive responses to the occurrence or anticipation of reward and termination of punishment. BIS is sensitive to signals of punishment and termination of reward, and is thought to control aversive motivation (Corr, 2008).

### Evidence of associations between trait-level BIS/BAS systems and alcohol misuse and alcohol use disorder

As alcohol use was shown to activate the brain reward system (Ingvar et al., 1998; Koob, 2003; Makris et al., 2008), individuals with strong BAS (high sensitivity to signals of rewards), may be more attentive to the pleasurable effects of alcohol than those with weak BAS. Accordingly, BAS sensitivity may increase the use of alcohol for positive reinforcement, and may constitute a risk factor for alcohol misuse. Studies investigating the link between the three different aspects of BAS sensitivity (i.e., fun seeking, drive, reward responsiveness) and alcohol use and misuse (Jorm et al., 1998; Loxton and Dawe, 2001; Franken and Muris, 2006; Booth and Hasking, 2009; O'Connor et al., 2009; Voigt et al., 2009; Hamilton et al., 2012; Wardell et al., 2012; Keough and O'Connor, 2014) showed a consistent, positive and significant association of the fun seeking aspect. However, as far as drive and reward responsiveness are concerned, results were mixed: associations were positive in some studies, negative or non-significant in others. This suggests that most of the influence of BAS in the development and maintenance of alcohol misuse comes from the fun seeking aspect. However, the role of drive and reward responsiveness remains unclear, and further studies are needed.

With regard to the link between BIS and alcohol use, two distinct paths may be expected. On the one hand, as BIS is related with enhanced anxiety and distress (Carver and White, 1994; Campbell-Sills et al., 2004), which are also associated with problematic drinking (Kuntsche et al., 2005), some researchers proposed that BIS sensitivity may lead individuals to drink in order to relieve their heightened anxiety and distress (e.g., O'Connor and Colder, 2005). In this perspective, high BIS may constitute an indirect risk factor for problematic drinking. It is not BIS *per se*, but rather the generated distress and anxiety that drive individuals to drink. On the other hand, as high-BIS individuals are more sensitive to signals of punishment than others, it may be that they overreact in face of the potential short-term negative consequences of drinking alcohol (e.g., hangover, accident), and that they therefore are more likely to avoid drinking. In this perspective, high BIS may constitute a direct protective factor for problematic drinking. Previous studies examining the relationship between BIS and alcohol use provided mixed findings: several studies failed to find a significant link between BIS and drinking behaviors (Jorm et al., 1998; Loxton and Dawe, 2007; Feil and Hasking, 2008; Loxton et al., 2008; Booth and Hasking, 2009; Lyvers et al., 2009; Keough and O'Connor, 2014), whereas others reported significant and negative (Loxton and Dawe, 2001; Franken and Muris, 2006; Pardo et al., 2007; O'Connor et al., 2009; Voigt et al., 2009; Wardell et al., 2012) or positive associations (Hamilton et al., 2012; Wardell et al., 2013). Moreover, when significant, the observed associations were weak. Thus, taken together, these results provided limited support for any of the hypothesized patterns of association. This lack of empirical support may arise from the inability of traditional regression models (used in almost all above-mentioned studies) to decompose the two hypothesized paths linking BIS to alcohol use behaviors. Indeed, positive and negative BIS–alcohol use behavior paths may cancel each other, resulting in null associations. A mediation analysis may provide a more detailed examination of the associations of alcohol misuse with BIS and BAS sensitivity than traditional regression models, as it allows to estimate the direct and indirect (i.e., mediated by more proximal factors) contributions of BIS and BAS sensitivity.

### Drinking motives may mediate the relationship between BIS/BAS and alcohol misuse and alcohol use disorder

Drinking motives (DM)—the value placed on the particular effects that individuals want to achieve when drinking alcohol (Cox and Klinger, 2011)—may constitute a relevant mediator accounting for the indirect associations of BIS and BAS sensitivity with alcohol use. Indeed, DM are often considered as the most proximal factors underlying drinking behavior, through which the influence of more distal factors such as personality traits is mediated (Cooper, 1994; Tragesser et al., 2007; Kuntsche et al., 2008). Two distinct dimensions are thought to underlie drinking motives: valence (i.e., drinking to enhance positive or reduce negative affect) and source (i.e., drinking to obtain an internal reward or to achieve external reward) of the outcome individuals hope to achieve by drinking (Cox and Klinger, 2011). By crossing the valence and source dimensions, four distinct drinking motives can be distinguished (Cooper, 1994). Social motives (e.g., drinking because it makes social gatherings more fun) reflect drinking for positive, externally generated reinforcement; conformity motives (e.g., drinking so as not to feel left out) denote drinking for negative, externally generated reinforcement; enhancement motives (e.g., drinking to get high) refer to drinking for positive, internally generated reinforcement; coping motives (e.g., drinking to forget worries) indicate drinking for negative, internally generated reinforcement. Furthermore, the four DM are differentially associated with alcohol use and misuse. Enhancement and coping motives are strongly related to alcohol misuse and alcohol-related consequences, whereas social and conformity motives are related to moderate alcohol use (Kuntsche et al., 2005; Graziano et al., 2012).

### Previous work on mediation of drinking motives and rationale for the current study

To our knowledge, only three studies investigated whether the associations of BIS and BAS sensitivity with alcohol use behaviors were mediated by drinking motives. In a sample of U.S. college freshmen, O'Connor and Colder (2005), showed that the association between BAS sensitivity and problematic drinking was mediated by enhancement, social and coping motives. However, this study has limitations in that it did not consider the three different aspects of BAS sensitivity (fun seeking, reward responsiveness, drive) separately, and only examined the direct (not the indirect) association of BIS sensitivity and problematic drinking. In a sample of 262 Australian adults, Lyvers et al. (2012) showed that the direct association between BIS sensitivity and risky alcohol use was negative, whereas BIS sensitivity was indirectly and positively associated via coping DM. This result supports the existence of two distinct paths linking BIS sensitivity with alcohol misuse in opposite directions. However, this study, too, has limitations in that it did not consider the potential mediation via other DM than coping, and only investigated the direct (not the indirect) association of BAS sensitivity. Finally, in a sample of 188 Belgian adolescents, Willem et al. (2012) showed that the positive association between the fun seeking aspect of BAS sensitivity and alcohol use was fully mediated by enhancement and social DM. However, they did not investigate the mediation of the associations of alcohol use and aspects of BAS other than fun seeking (i.e., reward responsiveness, drive) and BIS sensitivity.

**Figure 1:**
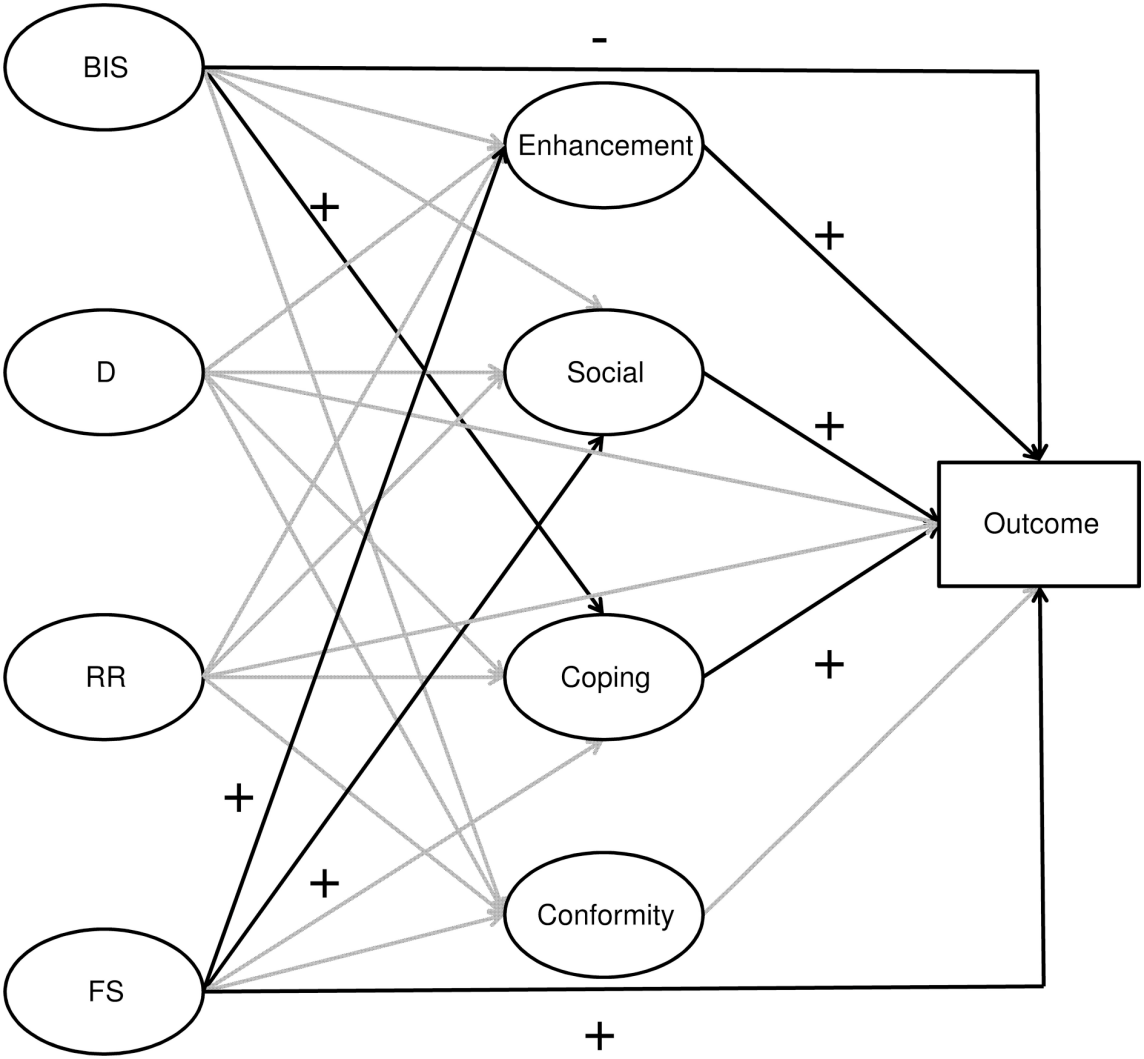
**Schematic representation of the hypothesized direct and indirect (via drinking motives) associations of Behavioral Inhibition System (BIS), Drive (D), Reward Responsiveness (RR) and Fun Seeking (FS) with drinking outcomes**. Black arrows denote hypothesized associations. Gray arrows denote associations with no a priori hypotheses.

Using a large representative sample of young Swiss men, the present study aimed at providing a more comprehensive picture of the associations of alcohol misuse and its negative consequences with BIS and the three aspects of BAS sensitivity (fun seeking, reward responsiveness, drive). It further sought to test whether the associations were mediated by DM. We expect that fun seeking aspects of BAS sensitivity will be positively associated with alcohol misuse (as measured by at least monthly RSOD) and alcohol use disorder, and that this association will be mediated by enhancement and social DM, the latter being associated with positive reinforcement. With regard to BIS sensitivity, we expect a negative direct association and a positive indirect association via coping DM, the latter being associated with general anxiety and tension reduction. As mixed results were found with regard to reward responsiveness and drive aspects of BAS sensitivity in previous studies, we cannot formulate specific hypotheses. Nevertheless, their direct and indirect associations with alcohol misuse and alcohol use disorder will be examined for exploration purposes. The hypothesized associations are illustrated in Figure 1.

## Materials and methods

### Study design and participants

We analyzed cross-sectional data from the first follow-up of the Cohort Study on Substance Use Risk Factors (C-SURF). C-SURF is a longitudinal study designed to investigate risk and protective factors of substance use in emerging adulthood. The research protocol (15/07) was approved by the ethics committee for clinical research of Lausanne University Medical School. Participants were enrolled in three of the six army recruitment centers in Switzerland, covering 21 of 26 Swiss cantons. As army recruitment is mandatory for 20-year-old males in Switzerland, virtually all young males of this age group were eligible for participation. Since questionnaires were completed at home, participants were not influenced by army procedures when filling out questionnaires. More information on enrolment procedure and non-consent and non-response bias was provided in previous studies (Studer et al., 2013a,b).

Since BIS/BAS sensitivity was only assessed in C-SURF's follow-up questionnaire, the present study focused exclusively on data from the follow-up questionnaire. During enrolment, a total of 7556 participants gave written consent to participate and, among them, 6020 (79.7%) completed the follow-up questionnaire between March 2012 and April 2013. Alcohol abstainers (*n* = 458, 7.6%) were excluded, because DM were only assessed among 12-month drinkers. Moreover, 200 participants (3.6% of 12-month drinkers) were excluded because they did not fill in the questionnaire assessing DM or BIS/BAS sensitivity. As a result, the analytical sample comprised 5362 participants (96.4% of 12-month drinkers).

### Instruments

French (Caci et al., 2007) and German (Strobel et al., 2001) translations of the BIS/BAS scales (Carver and White, 1994) were used to assess individual differences in BIS and BAS reactivity. This self-reported questionnaire comprised 20 items evaluated on a four-point scale ranging from 1 (“very true for me”) to 4 (“very false for me”). Items were recoded in such a way that high values were indicative of a higher level of endorsement of the item. A recent re-examination of the psychometric properties of the French and German versions of the BIS/BAS scales using the same sample (Studer et al., 2016) showed that nine items had poor loadings on their respective factors, and that their exclusion was needed to achieve satisfactory fit statistics. These items were also excluded in the current study. BIS (five items), drive, reward responsiveness and fun seeking (two items each) scales were treated as latent variables for ordinal data in the analyses, and mean scores for each scale were computed separately for descriptive purposes.

DM were assessed using the Drinking Motives Questionnaire Revised Short Form (DMQ-R SF; Kuntsche and Kuntsche, 2009). The DMQ-R SF consists of twelve statements, i.e., three for each of the four DM: social, enhancement, coping, and conformity. Using a five-point scale, ranging from 1 (“never”) to 5 (“always”), participants were asked to consider all the times they had drunk alcohol in the past 12 months, and to indicate the frequency at which they had drunk for each specific reason. The four DM were treated as latent variables for ordinal data in the analyses, and mean scores for each scale were computed separately for descriptive purposes.

The 11 criteria for alcohol use disorder (AUD) according to the fifth edition of the DSM-5 (American Psychiatric Association, 2013) were used to assess AUD at follow-up. Questions from Knight et al. (2002), as well as an additional criteria for craving were translated into French and German. Participants were asked whether they experienced each criterion in the previous 12 months. The sum of the criteria endorsed was computed and individuals were classified into four groups following DSM-5 cut-off for AUD severity: no, mild, moderate and severe AUD. For the analyses, a binary outcome was computed to differentiate between no/mild and moderate/severe AUD.

The frequency of RSOD was assessed by asking participants how often they had at least six standard drinks on a single occasion in the past 12 months. Possible answers were “never,” “less than once a month,” “every month,” “every week,” “every or nearly everyday.” A dichotomous variable “at least monthly RSOD” differentiating between monthly or more frequent RSOD (coded 1), and “less than monthly RSOD” (coded 0) was created. This variable was used as outcome in the analysis. As a sensitivity analysis, the original response scale was transformed into a number of RSOD episode in the previous 12 months (never coded 0, less than once a month coded 6, every month coded 30, every week coded 169, every or nearly everyday coded 286) and a logarithmic transformation was applied to approximate a normal distribution. Then, a linear model was fitted but results are not reported since they were approximately the same as those obtained with the binary outcome.

Socio-demographic variables including age, language and highest completed level of education, religious self-description, marital status, perceived family income were assessed. Highest completed level of education consisted of three categories of schooling: primary schooling (9 years); vocational training (>9–12 years); post secondary schooling (13 years or more, including high school, which can be only 12 years in some cantons). Language differentiated between French- and German-speaking participants. Religious self-description consisted of five categories: atheist, agnostic unsure, spiritual, and religious.

### Statistical analyses

**Figure 2:**
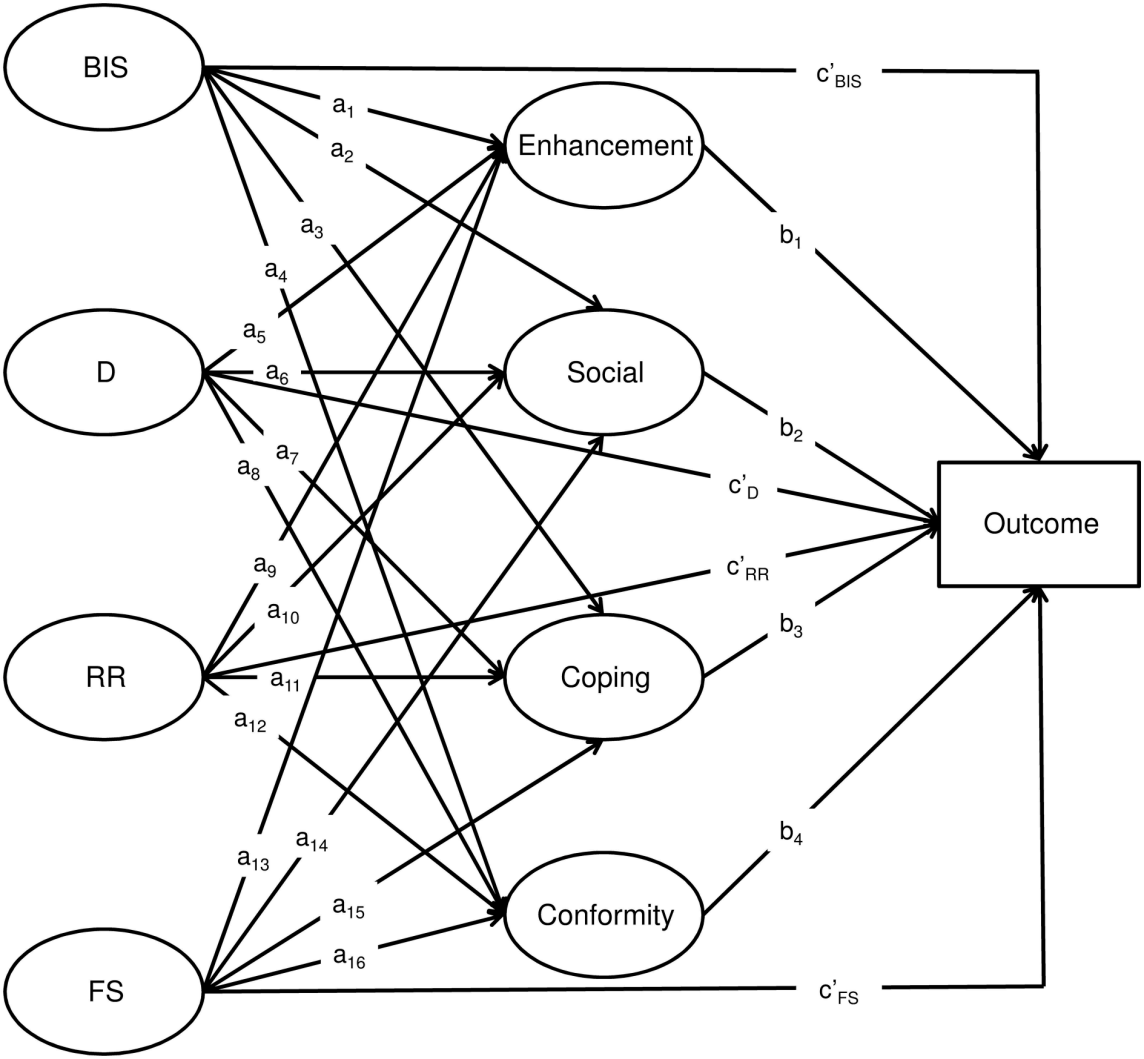
**Schematic representation of the structural model of Behavioral Inhibition System (BIS), Drive (D), Reward Responsiveness (RR), and Fun Seeking (FS) directly associated with drinking outcome, and indirectly via drinking motives**. Age, highest achieved education and linguistic region covariates and covariances between BIS, D, RR, FS and between drinking motives are not displayed merely for ease of presentation.

Descriptive statistics were calculated to characterize the sample in terms of age, linguistic region, highest completed level of education, at least monthly RSOD, AUD, DM, BIS, and BAS sensitivity. Zero-order correlations between all variables were used to examine the raw associations. Simultaneous associations of BIS, fun seeking, drive, and reward responsiveness with at least monthly RSOD and AUD, and the mediation via DM, were all tested using Mplus 7.11 (Muthén and Muthén, 1998-2013). Structural equation models (SEM) were calculated separately for AUD and at least monthly RSOD. Age, linguistic region and highest completed level of education were included as covariates in both models. Mediation analyses were computed following the procedure proposed by MacKinnon et al. (2007). To do this, SEM (see Figure 2 for a graphical representation of the structural model) estimate the paths (c′) between predictors (i.e., BIS, drive, reward responsiveness, fun seeking) and outcome, the paths (a_i_) between the predictors and mediators (i.e., DM), and the paths (b_i_) between mediators and outcome. The specific indirect association of a given predictor on the outcome, via a given DM, is defined as the product of the path linking that predictor to the DM (a_i_) and the path linking that DM to the outcome (b_i_). For example, the specific indirect association of BIS with the outcome, via enhancement DM, is quantified as a_1_ * b_1_ (see Figure 2). The total indirect association of a predictor with the outcome is the sum of all the specific indirect associations of that predictor. For example, in Figure 2, the total indirect association of BIS is defined as $\overset{4}{\underset{i=1}{\sum }}$(*a*_i_*b_*i*_). The total association (c) of a given predictor with the outcome is the sum of the direct association (c′) and the total indirect association. For example, for BIS, the total association is defined as $c=c\prime +\overset{4}{\underset{i=1}{\sum }}\left(a_{i}*b_{i}\right)$.

Parameter estimates were based on the weighted least squares mean-variance adjusted estimator (WLSMV), which was developed to handle ordinal indicators (Muthén and Muthén, 1998-2013). Bias corrected bootstrap 95% confidence intervals were computed by means of bootstrap resampling with 1000 draws. The statistical analysis handled missing values (0.10% of all data, 2.85% of participants with at least one missing value). The model fit was examined using the comparative fit index (CFI), the Tucker–Lewis index (TLI) and the root mean square error of approximation (RMSEA). While CFI and TLI higher than 0.95 and RMSEA of 0.06 or lower are indicative of good fit, values greater than 0.90 for CFI and TLI and values ranging between 0.06 and 0.08 for RMSEA are generally deemed acceptable (Browne and Cudeck, 1993; Hu and Bentler, 1999; Kline, 2011).

## Results

### Descriptive characteristics of the sample

The mean age of participants was 21.33 years (*SD* = 1.26). A little more than half of the participants (56.6%) were French-speaking, whereas 43.4% were German-speaking. With regard to highest achieved education, 7.5% of the sample reported primary schooling, 46.6% reported vocational training, and 45.9% reported post secondary schooling. Other socio-demographic characteristics and mean scores of DM and BIS/BAS sensitivity are reported in Tables 1, 2, respectively. Participants reported 1.32 AUD criteria on average (Median = 1.00, *SD* = 1.67). With regard to DSM-5 severity categories, 65.7% of the participants were classified as no AUD, 24.2% as mild, 7.2% as moderate and 3.0% as severe AUD. A little less than 48.1% of the sample reported at least monthly RSOD in the past 12 months.

**Table 1:** **Descriptive characteristics of the sample**.

	***N***	**%**
**RELIGIOUS SELF-DESCRIPTION**		
Atheist	1449	27.0
Agnostic	876	16.3
Unsure	638	11.9
Spiritual	1485	27.7
Religious	515	9.6
Missing values^a^	399	7.4
**MARITAL STATUS**		
Single	5006	93.4
In a relationship	288	5.4
Divorced	3	0.1
Married	56	1.0
Widowed	1	< 0.1
Missing values	8	0.1
**PERCEIVED FAMILY INCOME**		
Above average	2278	42.5
Average	2045	38.1
Below average	682	12.7
Missing values^a^	357	6.7
**LANGUAGE**		
French	3035	56.6
German	2327	43.4
**HIGHEST ACHIEVED EDUCATION**		
Primary schooling	404	7.5
Vocational training	2498	46.6
Post-secondary schooling	2460	45.9
**AGE**		
Age in years (Mean, SD)	21.33	1.26

*SD, standard deviation*.

a *This variable was assessed at baseline only*.

**Table 2:** **Means, standard deviation and range for drinking motives and BIS/BAS sensitivity scores**.

	**Mean**	**SD**	**Range**
**DRINKING MOTIVES**			
Enhancement	2.61	1.08	1–5
Social	2.79	1.08	1–5
Coping	1.63	0.79	1–5
Conformity	1.29	0.58	1–5
**BIS/BAS SENSITIVITY**			
BIS	2.65	0.57	1–4
Drive	2.26	0.75	1–4
Reward responsiveness	3.38	0.66	1–4
Fun seeking	2.54	0.66	1–4

*SD, standard deviation*.

### Associations with at least monthly RSOD and AUD and mediation via DM

Zero-order correlations between at least monthly RSOD, AUD, BIS, and BAS sensitivity and DM are reported in Table 3. The correlations between BIS/BAS sensitivity and alcohol use behaviors were of interest. Overall, magnitudes of these correlations were indicative of small effect size. BIS was significantly and positively associated with AUD, whereas it was negatively (although not significantly) associated with at least monthly RSOD. Reward responsiveness was significantly and negatively related to AUD, but positively (although not significantly) associated with at least monthly RSOD. Both drive and fun seeking were significantly and positively related to at least monthly RSOD and AUD, with larger coefficients of association for fun seeking than for drive and for AUD than for at least monthly RSOD. Almost all other correlations were positive and significant. The only exceptions were the significant and negative associations of reward responsiveness with coping and conformity DM and the non-significant and positive correlation between conformity DM and fun seeking. Interestingly, drinking motives were more strongly related to alcohol misuse than BIS and BAS sensitivity. Such an observation supports the idea that DM constitute the most proximal factors underlying drinking behaviors (Cooper, 1994; Tragesser et al., 2007; Kuntsche et al., 2008). Moreover, associations of coping and conformity DM were stronger with AUD than with at least monthly RSOD.

**Table 3:** **Zero-order correlations between alcohol use behaviors, BIS/BAS sensitivity and drinking motives**.

	**1**	**2**	**3**	**4**	**5**	**6**	**7**	**8**	**9**	**10**
1. At least monthly RSOD	–									
2. AUD	**0.55**	–								
3. BIS	−0.03	**0.10**	–							
4. D	**0.05**	**0.10**	**0.05**	–						
5. RR	0.01	−**0.08**	**0.37**	**0.28**	–					
6. FS	**0.19**	**0.23**	**0.24**	**0.61**	**0.45**	–				
7. Enhancement DM	**0.62**	**0.52**	**0.12**	**0.14**	**0.07**	**0.32**	–			
8. Social DM	**0.54**	**0.47**	**0.18**	**0.11**	**0.05**	**0.23**	**0.86**	–		
9. Coping DM	**0.36**	**0.48**	**0.17**	**0.16**	−**0.17**	**0.15**	**0.49**	**0.46**	–	
10. Conformity DM	**0.17**	**0.28**	**0.19**	**0.05**	−**0.26**	0.02	**0.37**	**0.43**	**0.57**	–

*Correlations in bold are significant at p < 0.05. RSOD, Risk single-occasion drinking; AUD, Alcohol use disorders; BIS, Behavioral inhibition system; D, Drive; RR, Reward responsiveness; FS, Fun seeking; DM, drinking motives*.

With regard to SEM of DM as mediators of the associations of BIS and BAS sensitivity with at least monthly RSOD and AUD, all fit indices were acceptable to good, which suggests an adequate fit of SEM for both at least monthly RSOD (CFI = 0.956; TLI = 0.944; RMSEA = 0.058) and AUD (CFI = 0.955; TLI = 0.943; RMSEA = 0.058). Results of these SEM are reported in Tables 4, 5. The total associations in Table 4 correspond to the simultaneous associations of BIS, drive, reward responsiveness and fun seeking predicting at least monthly RSOD and AUD adjusted for age, linguistic region and highest completed level of education, but without adjustment for DM. The total associations of the main outcomes at least monthly RSOD and AUD with drive and reward responsiveness were all significant (except for the association between AUD and drive) and negative, whereas the total associations of at least monthly RSOD and AUD with fun seeking were both positive and significant. Thus, the three subscales of BAS operated in different directions: drive and reward responsiveness were associated with less alcohol misuse or problems, whereas fun seeking was related to higher at least monthly RSOD and AUD. Moreover, as far as BIS is concerned, total associations with AUD and at least monthly RSOD ran in opposite directions: BIS was significantly associated with less at least monthly RSOD, but significantly associated with higher AUD.

**Table 4:** **Total, direct and total indirect associations of BIS and BAS sensitivity predicting at least monthly RSOD and AUD**.

	**Total association**			**Direct association**			**Total indirect association**		
	**β**	***b***	**95% CI**	**β**	***b***	**95% CI**	**β**	***b***	**95% CI**
**AT LEAST MONTHLY RSOD (***R*^2^ = 0.42**)**									
BIS	−**0.07**	−0.11	−0.18; −0.04	−**0.12**	−0.19	−0.27; −0.12	**0.05**	0.08	0.03; 0.13
Drive	−**0.08**	−0.12	−0.23; −0.02	−**0.07**	−0.10	−0.20; −0.01	−0.01	−0.02	−0.09; 0.05
Reward responsiveness	−**0.09**	−0.16	−0.27; −0.05	−0.01	−0.01	−0.12; 0.09	−**0.09**	−0.14	−0.22; −0.07
Fun seeking	**0.32**	0.50	0.37; 0.65	**0.09**	0.14	0.02; 0.27	**0.23**	0.36	0.28; 0.46
**AUD (***R*^2^ = 0.36**)**									
BIS	**0.11**	0.17	0.08; 0.28	0.03	0.05	−0.06; 0.15	**0.08**	0.13	0.08; 0.18
Drive	0.00	0.00	−0.13; 0.14	−0.02	−0.03	−0.18; 0.10	0.03	0.04	−0.02; 0.10
Reward responsiveness	−**0.28**	−0.45	−0.58; −0.33	−**0.15**	−0.24	−0.39; −0.09	−**0.13**	−0.21	−0.28; −0.14
Fun seeking	**0.32**	0.51	0.33; 0.72	**0.15**	0.24	0.06; 0.44	**0.17**	0.27	0.20; 0.35

*Beta coefficients in bold are significant at p < 0.05. BIS, behavioral inhibition system; RSOD, Risky single-occasion drinking; AUD, Alcohol use disorder; β, Standardized coefficient of association; b, Unstandardized coefficient of association; 95% CI, 95% bias corrected bootstrap confidence interval*.

**Table 5:** **Specific indirect associations of BIS and BAS sensitivity predicting at least monthly RSOD and AUD via drinking motives**.

	**DM enhancement**			**DM social**			**DM coping**			**DM conformity**		
	**β**	***b***	**95% CI**	**β**	***b***	**95% CI**	**β**	***b***	**95% CI**	**β**	***b***	**95% CI**
**AT LEAST MONTHLY RSOD**												
DM to at least monthly RSOD (b)	**0.46**	0.55	0.42; 0.68	**0.13**	0.16	0.04; 0.27	**0.18**	0.20	0.14; 0.27	−**0.14**	−0.16	−0.24; −0.09
**BIS**												
BIS to DM (a)	**0.05**	0.07	0.01; 0.12	**0.15**	0.20	0.14; 0.26	**0.27**	0.37	0.31; 0.44	**0.29**	0.39	0.33; 0.47
Specific indirect (a*b)	**0.02**	0.04	0.01; 0.07	**0.02**	0.03	0.01; 0.06	**0.05**	0.08	0.05; 0.10	−**0.04**	−0.06	−0.10; −0.03
**D**												
D to DM (a)	−0.03	−0.04	−0.12; 0.05	−0.01	−0.00	−0.08; 0.08	**0.17**	0.24	0.16; 0.33	**0.25**	0.30	0.21; 0.40
Specific indirect (a*b)	−0.01	−0.02	−0.07; 0.03	0.00	0.00	−0.01; 0.01	**0.03**	0.05	0.03; 0.07	−**0.03**	−0.05	−0.08; −0.02
**RR**												
RR to DM (a)	−**0.14**	−0.19	−0.26; −0.12	−**0.14**	−0.20	−0.28; −0.13	−**0.37**	−0.58	−0.66; −0.50	−**0.49**	−0.68	−0.78; −0.59
Specific indirect (a*b)	−**0.06**	−0.10	−0.16; −0.06	−**0.02**	−0.03	−0.06; −0.01	−**0.07**	−0.12	−0.16; −0.08	**0.07**	0.11	0.06; 0.17
**FS**												
FS to DM (a)	**0.39**	0.51	0.40; 0.63	**0.26**	0.35	0.26; 0.46	**0.14**	0.18	0.08; 0.30	0.04	0.06	−0.05; 0.18
Specific indirect (a*b)	**0.18**	0.28	0.20; 0.38	**0.04**	0.06	0.01; 0.10	**0.02**	0.04	0.02; 0.07	−0.01	−0.01	−0.03; 0.01
**AUD**												
DM to AUD (b)	**0.30**	0.36	0.16; 0.54	0.07	0.08	−0.09; 0.26	**0.30**	0.32	0.24; 0.41	−0.08	−0.09	−0.20; 0.01
**BIS**												
BIS to DM (a)	**0.05**	0.07	0.01; 0.12	**0.15**	0.20	0.14; 0.26	**0.26**	0.37	0.31; 0.44	**0.29**	0.39	0.33; 0.47
Specific indirect (a*b)	**0.02**	0.02	0.00; 0.05	0.01	0.02	−0.02; 0.05	**0.08**	0.12	0.09; 0.16	−0.02	−0.04	−0.08; 0.01
**D**												
D to DM (a)	−0.03	−0.04	−0.12; 0.05	0.00	0.00	−0.08; 0.08	**0.19**	0.24	0.16; 0.33	**0.25**	0.30	0.21; 0.40
Specific indirect (a*b)	−0.01	−0.01	−0.04; 0.02	0.00	0.00	−0.01; 0.01	**0.06**	0.08	0.05; 0.12	−0.02	−0.03	−0.06; 0.01
**RR**												
RR to DM (a)	−**0.14**	−0.19	−0.26; −0.12	−**0.14**	−0.20	−0.28; −0.13	−**0.39**	−0.58	−0.66; −0.50	−**0.49**	−0.68	−0.78; −0.59
Specific indirect (a*b)	−**0.04**	−0.07	−0.12; −0.03	−0.01	−0.02	−0.06; 0.02	−**0.12**	−0.19	−0.24; −0.13	0.04	0.06	−0.01; 0.14
**FS**												
FS to DM (a)	**0.39**	0.51	0.41; 0.63	**0.26**	0.35	0.26; 0.46	**0.13**	0.18	0.08; 0.30	0.04	0.06	−0.05; 0.18
Specific indirect (a*b)	**0.12**	0.19	0.08; 0.29	0.02	0.03	−0.03; 0.10	**0.04**	0.06	0.02; 0.10	−0.00	−0.01	−0.03; 0.01

*Beta coefficients in bold are significant at p < 0.05. BIS, behavioral inhibition system; D, Drive; RR, reward responsiveness; FS, Fun seeking; DM, drinking motives; RSOD, Risky single-occasion drinking; AUD, Alcohol use disorder; β, Standardized coefficient of association; b, Unstandardized coefficient of association; 95% CI, 95% bias corrected bootstrap confidence interval*.

The results of the mediation analysis, i.e., the decomposition of the total associations of at least monthly RSOD/AUD and BIS/BAS sensitivity in direct and indirect (mediated by DM) associations, are reported in Tables 4, 5. A significant and negative direct association was found between BIS and at least monthly RSOD, whereas significant and positive indirect associations were found via enhancement, social and coping motives, with a larger coefficient for the indirect association via coping than for associations via enhancement and social DM. In addition, a significant, negative and indirect association was found between BIS and at least monthly RSOD via conformity DM. A similar pattern of result was found in the indirect associations between BIS and AUD, except that indirect associations via social and conformity motives were not significant. Although, not significant, the direct association between BIS and AUD was nevertheless positive, as opposed to the direct BIS–at least monthly RSOD association. As for fun seeking, significant and positive, direct and indirect associations via enhancement, social and coping DM were found with at least monthly RSOD. A similar pattern of direct and indirect associations was found between fun seeking and AUD, except that indirect association via social motives was not significant. The size of the indirect associations of fun seeking was larger via enhancement than via social and coping motives. With regard to drive, results showed that direct associations with at least monthly RSOD and AUD were both negative, but that only the direct association with at least monthly RSOD was significant. Significant positive indirect associations were found via coping DM between drive and both at least monthly RSOD and AUD, whereas significant negative indirect association was found via conformity DM between drive and at least monthly RSOD. For reward responsiveness, negative and direct associations were found with both outcomes (only significant for AUD). Reward responsiveness was also indirectly associated with both at least monthly RSOD and AUD: negatively via enhancement, social (only significant for at least monthly RSOD) and coping DM, and positively via conformity DM (only significant for at least monthly RSOD).

## Discussion

The aim of the present study was to better understand the associations of personality traits related to appetitive and aversive motivations with alcohol misuse and problems in order to identify potential treatment and prevention targets. To do so, mediated associations by proximal factors related to appetitive and aversive motivation (i.e., DM) were tested. The present study provides both supporting and conflicting evidence to the hypothesis that BAS sensitivity is positively associated with alcohol misuse and alcohol problems. Supporting evidence was found by findings regarding only one aspect of BAS sensitivity, namely fun seeking. Fun seeking was positively associated with both at least monthly RSOD and AUD (total association in SEM), a finding in line with results of several previous studies (Jorm et al., 1998; Loxton and Dawe, 2001; Franken and Muris, 2006; Booth and Hasking, 2009; O'Connor et al., 2009; Voigt et al., 2009; Hamilton et al., 2012; Wardell et al., 2012; Keough and O'Connor, 2014). This suggests that fun seeking constitutes a risk factor for alcohol misuse and problems. More interestingly, results of the mediation analysis showed that a large part of the positive total associations of fun seeking were indirect and mediated mostly by enhancement DM and by social and coping DM to a lesser extent. These indirect associations via more proximal factors such as DM reflect the theoretical foundation of fun seeking, i.e., the willingness to approach a potentially rewarding event on the spur of the moment. This is particularly true for enhancement DM that are thought to underlie drinking to increase positive affect (Cooper et al., 1995). Consistent with previous studies (O'Connor and Colder, 2005; Willem et al., 2012), this finding suggests that high fun seeking individuals may be more prone to alcohol misuse and problems because they are more sensitive to the reinforcement properties of alcohol, which make them more likely to use it to enhance positive affect.

As previous studies showed mixed results with regard to the associations of alcohol misuse and alcohol problems with the two other aspects of BAS sensitivity, no specific pattern of associations was expected. Drive was negatively associated with at least monthly RSOD only, whereas reward responsiveness was negatively associated with both outcomes (see total association in SEM), suggesting that these aspects of BAS sensitivity constitute protective factors. With regard to drive more specifically, the negative association with at least monthly RSOD was only observed in SEM (adjusted for BIS and the two other BAS aspects), whereas bivariate association (zero-order correlations) yielded a positive association. The lack of consistency between bivariate associations and SEM probably arises from the substantive overlap between fun seeking and drive (*r* = 0.61), and may imply the presence of a suppression (see Maassen and Bakker, 2001, for more information about suppression). Leone and Russo (2009) investigated the associations between the three BAS aspects and functional i.e., the tendency to make quick decisions when it is beneficial, and dysfunctional impulsivity, i.e., the tendency to make quick decisions when it is not optimal and without considering the consequences of behaviors. Drive was significantly positively associated with both functional and dysfunctional impulsivity in the bivariate analysis, but only with functional impulsivity when adjusting for fun seeking (Leone and Russo, 2009). Thus, the results of the present study may indicate that high drive individuals may be less likely to misuse alcohol because it has more negative than beneficial consequences. Moreover, results of mediation analysis showed that only a small proportion of the negative drive–at least monthly RSOD association was mediated by DM. This suggests that the protective effect of drive is relatively independent of the contribution of DM. As for reward responsiveness, negative associations with at least monthly RSOD and AUD were only found in SEM (see total association). Although contrasting with the non-significant findings observed in several previous studies (Feil and Hasking, 2008; Booth and Hasking, 2009; O'Connor et al., 2009), this finding is in line with two previous studies showing a similar pattern of association (Voigt et al., 2009; Wardell et al., 2012). This suggests that as it is the case for drive, reward responsiveness may constitute a protective factor for alcohol misuse, but also a protective factor for AUD. As proposed by Voigt et al. (2009), one way to understand the opposing direction of associations of reward responsiveness and fun seeking may be to consider reward responsiveness as a construct linked to long term consequences, while fun seeking focuses on more immediate gratifications. Accordingly, individuals with high reward responsiveness may drink less than others because alcohol misuse is associated with more negative than positive long-term consequences. This proposition is also consistent with the stronger association observed between reward responsiveness and AUD (i.e., the negative long-term consequences of drinking alcohol) than between reward responsiveness and at least monthly RSOD. Moreover, results of mediation analysis suggest that most of the protective influence of reward responsiveness may be explained by the fact that individuals reporting high reward responsiveness use less coping and enhancement DM than others.

Taken together, results suggest that it is important to consider each aspect of BAS sensitivity in alcohol studies because fun seeking, drive and reward responsiveness are differentially associated with alcohol misuse and problems. Moreover, further studies should be conducted in order to better understand the mechanisms underlying the association of each aspect of BAS sensitivity, in particular for those for which protective effects were observed (i.e., drive and reward responsiveness).

As for BIS, only findings regarding at least monthly RSOD provided support for the hypothesized negative direct association and positive indirect association via coping DM. In line with some previous studies (Franken and Muris, 2006; Pardo et al., 2007; Wardell et al., 2012), the total association with at least monthly RSOD was negative and mostly direct, as shown in the mediation analysis. Since the most common negative consequences of RSOD include the acute physiological and behavioral effects of excessive alcohol use such as blackouts, regretted actions, violence, accidents or injuries (Wechsler et al., 1994; Daeppen et al., 2005; Kuntsche and Gmel, 2013), this finding supports the idea that individuals with high BIS are more prone than others to avoid risky drinking, because their heightened sensitivity to signals of punishment make them more aware of the short-term negative consequences of drinking alcohol. Although small, positive indirect associations were also found via DM (mostly coping). This latter finding supports the idea (O'Connor and Colder, 2005) that BIS predisposition to enhanced anxiety and distress may also urge some individuals to misuse alcohol so as a way to regulate their negative emotional states (via coping DM). In line with previous results (Lyvers et al., 2012), findings regarding AUD further supported this proposition: total association between BIS and AUD was positive and this association was fully mediated by DM (mostly by coping DM). By contrast, contrary to our expectations, no evidence of a direct negative BIS-AUD association was found. This is probably because AUD reflect more long-term problems than RSOD.

Taken together, the differential associations of BIS with RSOD and AUD suggest that BIS constitutes a protective factor for RSOD and risk factor for AUD. The negative direct association and the positive indirect associations of BIS with RSOD may reflect the fact that RSOD is associated with short-term consequences but also with more long-term problems such as AUD (Knight et al., 2002; Baggio et al., 2015), whereas AUD reflects more long-term problems only.

This study is not without limitations. First, the mediation analysis assumes a temporal ordering of the variables: the predictors (i.e., BIS/BAS sensitivity) should precede the mediators (i.e., DM) and the outcome variables (i.e., at least monthly RSOD and AUD). This assumption was not tenable in the present study, as the design was cross-sectional. However, this should not be a problem, given that personality traits reflect the expression of genetically-determined systems (Eysenck, 1990) that are relatively stable over time (McCrae and Costa Jr, 1994). Accordingly, BIS/BAS sensitivity precedes DM and alcohol misuse and alcohol problems. Nevertheless, further research should be conducted to replicate the findings of the present study, this time using a longitudinal design. Second, the sample was confined to young adult males, thereby preventing the results from being generalized to females or people in other age ranges.

To conclude, results showed that among the different aspects of BAS sensitivity, only the predisposition to fun seeking constitutes a relevant risk factor for alcohol misuse, and that most of the contribution accounted for by fun seeking is mediated by enhancement DM. Similarly, BIS constitutes a risk factor that is mainly mediated by coping DM. However, this mediated association dominated the total association with BIS only for AUD, which can be seen as consequences in the long run, and a long-term reaction to regulate the general anxiety and distress generated by high BIS. For more short-term effects such as at least monthly RSOD, the BIS total association is dominated by the negative direct association, thereby supporting the idea that, because BIS individuals are more sensitive to signals of punishment than others, they may overreact in face of the potential negative consequences of drinking alcohol (e.g., hangover, accident), and, for this very reason, avoid drinking. In line with evidence suggesting that prevention programs targeting DM may be effective in reducing alcohol misuse associated with at-risk personality traits (e.g., Stewart et al., 2005; Conrod et al., 2006, 2013), the results of the present study suggest that intervention should focus on different DM in order to reduce the risk associated with high fun seeking and the predisposition to high BIS. Intervention targeting the enhancement DM, such as providing alternative sources of stimulation, promoting attractive and alternative activities instead of drinking alcohol, or altering the expectancies about the enhancing effects of alcohol (Cooper et al., 1995; Urbán et al., 2008; Correia et al., 2011) may be particularly effective in reducing the risk associated with high fun seeking. By contrast, intervention targeting coping DM, such as reducing levels of stress, providing alternative ways of coping than drinking alcohol, and by building self-esteem and competencies by means of life skills training (Cooper et al., 1995; Botvin, 2000; Kuntsche et al., 2010; Németh et al., 2011) may be particularly effective in decreasing the risk associated with the predisposition to high BIS. Moreover, since recent studies showed that training of alcohol approach/avoidance tendencies may be effective to reduce alcohol use (e.g., Wiers et al., 2010, 2011), results of the present study suggest that assessment of enhancement and coping DM, as well as BIS/BAS sensitivity may be useful to better identify individuals for whom such training interventions would be the most effective.

## Author contributions

GG, JD, and MM conceived and designed the study. JS, GG contributed to the acquisition of the data and conducted the analyses. All authors contributed to the interpretation of the data for the work. JS drafted the manuscript. GG, JD, MM, SB, and MD critically revised the manuscript. All authors approved the final version of the manuscript to be published and agreed to be accountable for all aspects of the work in ensuring that questions related to the accuracy and integrity of any part of the work are appropriately investigated and resolved.

## Funding

This study was funded by the Swiss National Science Foundation (FN 33CSC0-122679 and FN 33CS30-139467). We are grateful to Charlotte Eidenbenz for her extensive efforts in the coordination of this study and English editing.

### Conflict of interest statement

The authors declare that the research was conducted in the absence of any commercial or financial relationships that could be construed as a potential conflict of interest. The reviewer, MK and handling Editor declared their shared affiliation, and the handling Editor states that the process nevertheless met the standards of a fair and objective review.
